# Chronic Wasting Disease (CWD) in Sami Reindeer Herding: The Socio-Political Dimension of an Epizootic in an Indigenous Context

**DOI:** 10.3390/ani11020297

**Published:** 2021-01-25

**Authors:** Simon Maraud, Samuel Roturier

**Affiliations:** Université Paris-Saclay, CNRS, AgroParisTech, Ecologie Systématique Evolution, 91405 Orsay, France; samuel.roturier@universite-paris-saclay.fr

**Keywords:** chronic wasting disease (CWD), governance, reindeer husbandry, Indigenous local knowledge (ILK), health risk management

## Abstract

**Simple Summary:**

Chronic wasting disease (CWD), the most transmissible of the prion diseases, was detected in 2016 in Norway in a wild reindeer. This is the first case in Europe, an unexpected one. This paper focuses on the issues that the arrival of CWD raises in Northern Europe, especially regarding the Indigenous Sami reindeer husbandry in Sweden. The study offers a diagnosis of the situation regarding the management of the disease and its risks. We present the importance of the involvement of the Sami people in the surveillance program in order to understand better the diseases and the reindeer populations, movement, and behavior. However, the implementation of new European health standards in the Sami reindeer herding could have tremendous consequences on the evolution of this ancestral activity and the relationship between herders and reindeer.

**Abstract:**

Chronic wasting disease (CWD) is the most transmissible of the prion diseases. In 2016, an unexpected case was found in Norway, the first in Europe. Since then, there have been 32 confirmed cases in Norway, Sweden, and Finland. This paper aims to examine the situation from a social and political perspective: considering the management of CWD in the Swedish part of Sápmi—the Sami ancestral land; identifying the place of the Sami people in the risk management–because of the threats to Sami reindeer herding that CWD presents; and understanding how the disease can modify the modalities of Indigenous reindeer husbandry, whether or not CWD is epizootic. Based on interviews with various stakeholders and by examining the social sciences literature, this paper shows that the health risk management is structured by a politico-scientific controversy about the recognition, or not, of atypical and classical CWD. The Sami herders are currently cooperating with the state authorities in the surveillance program to sample their herds. This involvement takes place in a situation where the balance of power between the Sami people and the state, or the European Union, is framed by its colonial context. This has consequences with respect to the definition of a common interest and to implementing sanitary measures. The particular features of reindeer herding are seen as a challenge to managing CWD risk, compared with European health standards. We argue that CWD will greatly modify the modalities of Indigenous reindeer herding, whether there are positive cases or not in the Sami reindeer. By implementing new health guidelines, the authorities will create a cascading effect in Sami land and its use. The CWD situation in Fennoscandia is full of uncertainty but may cause a major shift in the organization and the governance of Sápmi. In September 2020, the identification of a new CWD case in a wild reindeer in Norway started a new episode in the disease management in Fennoscandia. Our paper raises various questions linked to understanding this new step in this crisis which is not only epidemiological, but also socio-cultural and political.

## 1. Introduction

Chronic wasting disease (CWD) is the most transmissible of the prion diseases [1,2]. It affects cervids (*Cervidae*) in captivity or in the wild, a rather uncommon situation among prion diseases [3]. The infectivity is located in muscles, antler velvet, endocrine glands, urine, saliva, blood, or feces. The prions affect other cervids’ lymphoid tissues and central n systems (CNS) [2]. It was detected for the first time in the 1960s in the United States of America, and has now spread to over 24 states of the US [2,4,5,6,7]. Many cases have also been found in Canada (with an outbreak in wild cervids) and a few in South Korea (in captive cervids only and CWD is now eradicated) [8]. In 2016, an unexpected case was found in Norway, the first in Europe, and a further 28 positive cases have been found in Norway, Sweden, and Finland since then, including a new case in a second population of wild reindeer in Norway in September 2020 [9]. However data coverage is rather poor in Fennoscandia, and most knowledge originates from North America [10]. In the literature, CWD has mostly been investigated and understood through a biological and epidemiological approach, with less social and even less political consideration (however, studies on social impacts of CWD exist with a focus on North America, e.g., [11,12,13,14,15,16]. This might be explained by the facts that it affects remote areas and because the position of cervids in the international market is low [3]. However, in the circumpolar North, CWD could have a major social, cultural, and political impact since many Indigenous people depend on *Rangifer* species for their livelihoods (the reindeer meat remains an important food source for the herders’ communities). In Fennoscandia 500,000 reindeer (*Rangifer tarandus tarandus*) are herded by Indigenous Sami people.

Social sciences can make a contribution to our understanding of the issue by examining the impact of infectious animal diseases and zoonoses on human societies. For instance, many studies taking a social science approach refer to bovine spongiform encephalopathy (BSE) or avian influenza [17,18,19,20,21,22], probably because of the established zoonotic potential and their clear impacts on social and economic structures. In the biological sciences, there are fewer studies of the CWD situation in Europe compared to North America, mainly due to its recent emergence. Depending on the zoonotic potential of CWD, this disease could have tremendous consequences among Indigenous populations because of their traditional activities and their potentially high exposure to the prion [14]. However, to our knowledge, no published study has highlighted this matter in Europe.

In this paper, we investigate the extent to which CWD is a socio-cultural and political crisis and not just a health crisis in Northern Europe. In addition, we examine the threats of CWD in Indigenous reindeer herding in Sápmi, the land of the Sami people—with a focus on Sweden. Our analysis has three main objectives: (i) to diagnose the situation and the management of CWD in Fennoscandia; (ii) to identify the place of the Sami people in the risk management; (iii) to understand how this phenomenon could modify the modalities of Indigenous reindeer herding, whether or not CWD is epizootic.

Our results are by no means recommendations, but an invitation to the researchers and various stakeholders concerned to take up these issues and be willing to contribute to a disciplinary broadening of the study of CWD. This project allows questions to emerge based on a social approach, and before an emergency situation is reached. This work covers the period from March 2016 (the discovering of the first case of CWD in Europe) to August 2020. The new case in wild reindeer in Norway in September 2020 will probably trigger a new episode in the management of CWD in Europe and reinforces the need to introduce social issues into CWD management. By examining these issues and focusing on their socio-political aspect, we hope to contribute to the body of knowledge about CWD and to support the implementation of tools to fight against it. Although our work adds an extra layer of complexity to understanding the disease and managing it, it offers many opportunities to discuss an epizootic in Indigenous contexts. We specifically discuss Sami reindeer herding, which is currently under particular threat, but the issues are likely to be relevant to other Indigenous people in subarctic regions.

## 2. Methods

### 2.1. Study Design

This study was developed on a two-step basis: a review of the literature on CWD in the biological sciences and on animal diseases in social anthropology; and ‘fieldwork’ based on semi-structured interviews. This qualitative study has been conducted using a multi-site and multi-scale approach (international, European, national, regional, and local), with a focus on Sweden due to our in-depth knowledge of reindeer herding in the area [23].

We conducted 20 interviews with three categories of stakeholders: institutional actors, scholars (veterinarians), and Sami reindeer herders (Table 1). During these interviews, we examined several topics: the discovery of CWD in Northern Europe; the strategy of the authorities for managing this situation; the tools used for management; the culling strategy; the role of the Sami herders in the management; the possibility and consequences of there being cases in reindeer husbandry; Sami knowledge and values related to dealing with epizootics in their activities; and the evolution of the situation in the future. They were all conducted remotely between May and July 2020, i.e., during the COVID-19 lockdown in Europe. Studying this issue during the implementation of measures targeting COVID-19 had an influence on our work by making any planned fieldwork impossible.

### 2.2. Ethics Statement

An important part of these analysis is based on interviews results. The interviewees were voluntary, and their answers were confidential and anonymized in this project. Participants made a verbal consent to contribute to this study according to these conditions. Written consent was not requested to facilitate the interaction between the interviewees and us, in an already complicated situation because of the lockdown and the cancelled fieldtrip. All the participants agreed to be recorded. This project and its method were approved by the Data Protection Officer of Paris Sud University (France) and the study was made in accordance with the GDPR.

### 2.3. Sami Reindeer Husbandry Context

**Figure 1:**
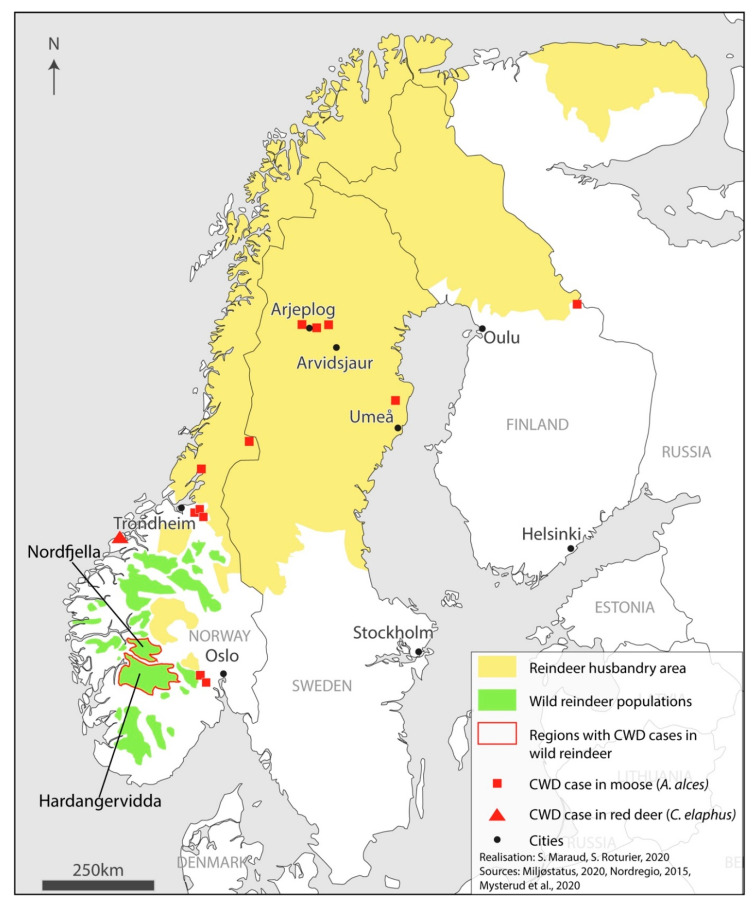
Map of the chronic wasting disease (CWD) risk in the reindeer populations in Fennoscandia. Sources: Villrein.no for wild reindeer populations; Nordregio (2015) for reindeer husbandry areas; Mysterud et al. (2020) for CWD cases.

The Sami are the only recognized Indigenous people of continental Europe. They are estimated to number 80,000 individuals. The Sápmi covers the northern regions of Norway, Sweden, Finland and the Kola peninsula in Russia (Figure 1). Although there may have been interaction between the Sami and the Nordic settlers before, it is considered that colonization started in the 16th century. This long and progressive process took place in subsequent centuries and accelerated in the 19th, and was based on racial hierarchies and competition between traditional Sami activities (hunting, fishing, and reindeer herding) and settlers’ agriculture and forestry [24,25]. In addition to assimilation and racist policies towards the Sami, reindeer husbandry progressively became the symbol of an essentialized Sami culture (however the great majority of Sami people never were and are not currently reindeer herders). In Sweden, the state organized this activity on the basis of reindeer herding communities. These communities (called ‘*sameby*’ in Swedish) are geographical units, with a limited number of reindeer allowed, which are determined by reindeer herders’ associations. It is mandatory to be a member of a community to practice reindeer husbandry and to benefit from the ancestral rights of the Sami. The form of pastoralism evolved considerably through history. For about a century, it took the form of extensive herding with alternating periods of loose and tight control, punctuated by seasonal operations to gather animals and structured by migrations between winter and summer grazing lands. Having free-ranging animals involves herds that mix with each other, sometimes crossing herding communities or national boundaries, but also with other wild animals.

## 3. Results and Discussion

### 3.1. Expansion of Chronic Wasting Disease

CWD is an epidemic of cervids, generating great concerns worldwide because of its contagious transmission in wild and captive animals in specific geographic areas–however new scientific works highlight the discovery of a sporadic CWD with other features and that is not epidemic [2,4,5,26,27]. It is a prion disease, or transmissible spongiform encephalopathy (TSE), like BSE or scrapie. Prion diseases affect the central nervous system (CNS) of mammals causing neurodegenerative disorders [2,6]. They are fatal and incurable [4]. The clinical signs of CWD usually appear in individuals older than 16 months, with an incubation period from two to four years, and in most cases death occurs within 4 months of the onset of the clinical disease [2]. CWD has the particularity among prion diseases to be highly contagious [4]. The prion is found in saliva, blood, urine, and stools and also in the peripheral lymphoid system which strengthens its transmission efficiency [2,28,29,30,31]. It has the ability to survive and contaminate the environment (via absorption of the prion by plants for instance), which remains a huge challenge for the management of the disease [28,32,33,34,35,36,37].

### 3.2. North America

CWD was first discribed in 1967 in mule deer (*Odocoileus hemionus*) and black-tailed deer (*O. hemionus columbianus*) in captive population, in Northern Colorado, US [2,8]. A few years later, it was found in Wyoming, Nebraska, and South Dakota [2,5,6]. A hypothesis is that older cases existed in wild cervids decades before [26].

According to the US Centers for Disease Control and Prevention, as of August 2020, there were cases in free-ranging cervids in at least 24 states. The expansion of CWD seems to be irreversible and it has spread progressively into new territories in captive and free-ranging populations due to animal migrations and herd movements and transportation [2]. The management of the disease in the country is challenging because of the high prevalence in wild populations and the lack of people working on this particular issue at the federal level (pers. comm., interview 4). The origin of CWD in the United States remains unknown [2].

### 3.3. Expansion of the Disease

Controlling the progression of an epizootic is highly complex, because of animal import/export across continents, and simply because political and administrative borders are largely porous to animals. A study shows that the first identification of CWD outside the USA was in a zoo in Toronto, Canada, from 1973 to 2003 [38]. At the end of the 20th century, the authorities had identified a case in Saskatchewan, Canada, in an elk (*Cervus canadensis*) imported from the US [7] and more cases were discovered during the following decade. In the 2000s, the first case was identified outside North America, in South Korea. The infection was due to the importation of infected animals from Canada [39]. In 2013, a moose (*Alces alces*) found on a road in Alberta, Canada, tested positive for CWD [2]. The disease is spreading in new wild animals (in Alberta and Saskatchewan) as in cervid farms [2]. In 2016, a case in Nordfjella, central Norway (Figure 1), was a major surprise. It was the first case in Europe, with no known connection with North America, and in a wild reindeer population (the first infected reindeer in North America was found later in a captive facility, in 2018) [2,8,40,41]. In 2018, Quebec, identified the first case of CWD in captivity and became the third Canadian province with infected cervids.

### 3.4. Nordfjella, a Dramatic Shift in Europe

In 2016, during a project on nomadic wild animals conducted by the Norwegian Institute for Nature Research, researchers found a sick reindeer that died in front of them (pers. comm., interview 1). Even though nobody expected that CWD was going to be found, they respected all the procedures during the postmortem and the analysis of the animal’s brain revealed that it was positive for this disease. As a result, a survey program was initiated. In the following months, two moose tested positive for CWD and another one was detected in 2017, but the prions were restricted to CNS [42]. The biochemical features of the prion found associated with moose were different from the cases in North America and Nordfjella—even though the strains found in Nordfjella also differ from the North American ones (but the prions were located in both CNS and lymph nodes) [2,4,8,42]. Between March 2016 and August 2020, 27 cases of CWD were detected in Norway: 19 wild reindeer from Nordfjella, 7 moose, and one wild red deer (*Cervus elaphus*) [10,27]. So far, the origin of CWD in Norway remains unknown [2,40].

In 2017, the Norwegian authorities made a drastic decision: to cull all the 2000 wild reindeer between 2017 and 2018 in the Nordfjella region to sample them and to limit the spread of the disease, especially towards the semi-domesticated reindeer in surrounding regions [2,8,10,40,43]. According to a veterinarian from the Norwegian Veterinary Institute, Norway was ‘lucky’ to find the case of CWD but also fortunate that it happened in Nordfjella because it is an area with natural borders, so without many animal movements with other regions around:

“I think we were very lucky that it was in that area, because the worst-case scenario would be to have the same case but in northern Norway. Because there, it’s impossible to trace where the animals have been, what the contacts have been, the borders are non-existent, the [Sami], on one side or the other, whether it’s Norway or Sweden, they don’t follow the borders, the animals go in summer on one side, in winter on the other.”

(pers. comm., interview 1)

However, Nordfjella is considered to represent a key step in the risk management of CWD in Europe, and in Fennoscandia. The slaughter of 2000 animals (between 2017 and 2018) was a debated, though radical, decision that actually impressed many reindeer herders, nature managers, scholars, and even the public. We will see later that it became one of the defining elements of the CWD issue in Europe.

Nordfjella triggered an increased survey of CWD in Northern Europe. It led to the identification of new cases in Norway, with the four additional cases mentioned earlier, but also in Finland with one moose in 2018, and four infected moose in Sweden in 2019, and one in September 2020 [10] (Figure 1). These cases kept adding to the complexity of the research on CWD in Europe because of the distances between them, which demonstrate that there was not only one cluster in Norway but probably several, related to the locations where the cases were identified [8].

The event in Nordfjella has created great fear among the Nordic countries and the European Union (EU). Knowledge of CWD was based on North American cases and much needed to be done in Europe to investigate since some features of the disease seemed to differ.

### 3.5. Uncertainty about a New Disease in Europe

According to a report from the European Food Safety Authority (EFSA) there are 13 groups of risk factors that may contribute to the spread of the disease [10]. Some are human-mediated and are thus considered preventable, others may contribute to the spread of the disease mainly via environmental contamination following death. Based on these risk factors, the European Commission started a surveillance program for CWD in 2018 for three years, to be implemented in six member states with moose or reindeer populations: Sweden, Finland, Estonia, Latvia, Lithuania, and Poland [10]. It had two goals: to gain an overview of the situation regarding CWD prevalence in EU, and to contribute to the research on this prion disease and study the potential zoonotic risk.

The method of the surveillance is to sample dead cervids and to send the samples to laboratories for testing. One Swedish veterinarian described a major aspect of the uncertainty there is in understanding CWD: “We haven’t got a clue about how long this disease has existed… what we do know is that we need to sample to find it” (pers. comm., interview 3). The clinical signs are not sufficient to identify the disease because they are not specific and they appear quite late in the development of the disease in the host [2]. Sampling wild animals is challenging because the chances of a sick animal in such areas being observed, reported and submitted for testing are extremely small [40]. Sixty per cent of the 6000 cervids tested were from healthy hunted/slaughtered fit for human consumption animals, whose probability of disease is lower than that of sick animals, road kill, or fallen stock (11). However, that figure varies between countries, as well as the species sampled and their general investment in the sampling program [10].

Infectious diseases are particularly challenging when attempting to implement coordinated mobilization because heterogeneous stakeholders need to be gathered rapidly “on the basis of limited knowledge” and “around an uncertain problem” [19]. This is why coordination is a very important but difficult step in managing CWD. The diversity of positions between the Nordic states may have an impact on the results of the survey:

“This is typically what happened in the United States. Each state decides what action to take. So, there have been states where they have actually slaughtered everything that moved in a radius around the cases, and when they get to the border, there is nothing left, and on the other side, no action.”

(pers. comm., interview 1)

Encouraged by the EU, Norway–a non-EU member state, but with the highest number of wild and domestic reindeer–took the lead in CWD surveillance among the European countries, with an overwhelming majority of tests [10]. Since 2016, the country has tested more than 100,000 cervids (pers. comm., interview 1). The program is taking place all over Norway and involves all cervid species. It is intensified in regions where CWD cases have been found, with the participation of the locals (pers. comm., interview 1). The hunters are equipped with sampling kits and play a key part in the sampling process by killing the animals and sending in the sample. The advanced approach of Norway made its results a benchmark for CWD knowledge in Europe. The Norwegian strategy impresses a lot of other countries and the EU because of its efficiency, but remains very difficult and uncertain because of the vastness of the territory and the complexity of identifying a prion in the environment (pers. comm., interviews 4, 5, 19).

In Sweden, the surveillance started later because the authorities decided to await the decision of the EU in relation to general surveillance (pers. comm., interview 3). On the Norwegian side, the position taken by Sweden was not understandable to one veterinarian interviewed: “what is surprising is that Sweden, in general, does the same as us […], they usually react very quickly to this kind of thing, and here, really, they didn’t live up to it” (pers. comm., interview 1). In Sweden, this slow reaction was controversial as well. For instance, a veterinarian from SVA explained that the surveillance started too late because it was not prioritized by the government of Sweden–despite the scientific recommendations (pers. comm., interview 3). This situation may be due to a complex administrative organization involving the Swedish Board of Agriculture (SBA) that takes the decisions and finances the surveillance, the Swedish Veterinary Institute (SVA), responsible of the analysis of the samples, and the Swedish Environmental Protection Agency (SEPA) that advices the SBA on long-term management plans for wild animals (pers. comm., interviews 3, 10, 17).

In Finland, according to the Ministry of Agriculture, there is a goal of 3000 samples, that they wish to reach by the end of 2020 (pers. comm., interview 15). However, the situation is different from Norway and Sweden: in Finland reindeer husbandry is carried out in fenced grazing areas. In other words, the risk of mixing populations is lower, and the management of a potential case in a reindeer herd could be easier (although this not the case with wild cervids).

Four years after its identification in Europe, Chronic Wasting Disease remains one of the most enigmatic prion diseases. Sampling is the main strategy implemented at the regional scale because of the continuing uncertainty about its various features, and much needs to be done in order to understand the differences and the similarities with North American CWD and the transmission of the disease.

### 3.6. Controversy of the Two CWDs and the Science–Policy Relationships

This section does not aim to analyze the sampling strategy for CWD in Europe from an epidemiologic perspective, but to examine the political dimension of the surveillance of an emerging epizooty. We found that knowledge about CWD does, indeed, have multiple political biases due to the variety of stakeholders involved and the multiple layers of political organizations. This clearly influences the funding of research on CWD and the priorities in CWD management between countries.

As Morand [44] pointed out: “Any analysis of temporal epidemiological trends must take into account the means that a country, or the international community, will put in place to monitor epidemics and to accurately characterize circulating or emerging pathogens”. Knowing the difference in the number of samples between Europe and North America is critical to understanding the current prevalence of CWD across the world and the fact that it is probably underestimated [2,10]. For instance, all the veterinarians we interviewed (from the US, Nordic countries or the EU) agreed that we need to know more. The results of surveillance inherently depend on the intensity of sampling. A more intensive sampling process would probably mean more CWD cases, in particular in older moose (pers. comm., interview 1). For now, the figures used by the Nordic authorities to describe the situation at the national level are too low to give a real picture of the CWD in the country (pers. comm., interview 3), and this is a political aspect since it is correlated to funding. In other words, the lack of data is above all a lack of investment.

The discovery of CWD in Europe shifted understanding of the disease and started what we can see as a science-policy controversy. The research conducted in Norway led to the identification of four prion strains: one identified in reindeer, one in red deer, and two in moose, all different from the ones found in North America (pers. comm., interview 1) [2,8,45]. CWD in moose and red deer was only found in old individuals. In addition, according to the veterinarians from Norway and Sweden, the disease seems to be less contagious in the European moose population than was observed in North America, even though the data regarding American moose are “sparse and insufficient to understand the epidemiology” for this species [8]. The findings led to a major hypothesis regarding the CWD found in the moose and red deer, namely that there could be two types of CWD: one where prions are only found in the CNS, not in the lymphatic system, spontaneous and sporadic (transmissible but not contagious), found in these moose and red deer, which could be an ‘atypical’ CWD; and another one with prions in the CNS and the lymphatic system, highly contagious, which could be the ‘classical’ CWD found in North America and Nordfjella (pers. comm., interviews 1, 3, 15, 17) [27].

This hypothesis has been central for the positioning of Sweden and Finland. The veterinarian from SVA said

“We knew from Norway that they already had these cases in old moose. When they found their first cases in moose, it was like ‘Oh my god, it is spread all over the country!’, but when we got our first case, it was like ‘Yeah, right, but this seems to follow another pattern’.”

(pers. comm., interview 3)

A scenario with cases in reindeer would probably have changed the behavior of Sweden and Finland (pers. comm., interview 1). However, identifying moose cases in the two countries led them to relativize the anxiety about the situation: according to the SBA, “there is nothing that points in the direction that we have something similar to what they have in Norway, so it is actually not so surprising that we have different systems for surveillance” (pers. comm., interview 17). For the authorities, the idea is thus not to overreact (pers. comm., interviews 15, 17). What makes it controversial is that the EU does not recognize the hypothesis of a ‘classical’ and an ‘atypical’ CWD. The EFSA explains that “[c]urrent data do not allow any conclusion on the implications of strain diversity on transmissibility (including host susceptibility, pathogenis and prevalence” [10]. For now, the European Union’s position is thus to consider the classical and atypical CWD dichotomy as a political discourse and not a scientific one (pers. comm., interview 19). According to the EU, to act differently whether the case is considered as ‘atypical’ or ‘classical’ could be a risky choice because of the lack of knowledge about what this variation implies in relation to prevalence, transmission, infection of the environment, or pathogens [10] (pers. comm., interview 19). For the interlocutors we questioned at the EU Commission, “local authorities and farmers should be more concerned” (pers. comm., interview 19). The two CWDs controversy reveals a situation that is considered to be serious yet with a lack of scientific consensus, mainly due to the lack of data. Hence the management of the health crisis is implemented by various stakeholders at various scales—states, veterinarian agencies, even Indigenous herding organizations—who seize the scientific hypotheses and results to inform their management decisions. By doing so they take part in the debate which has become a science-policy controversy.

The EU and its institutional functioning are not exempt from that process. The Commission actually depends on political moves to operate in CWD risk management at the European level, i.e., including the 27 member states. For example, red deer are present all over the EU, and one was found positive for CWD in Norway. However, this did not prompt general surveillance in every member state. According to our contacts at the EU Commission, “if we say: ‘We had a case in Norway, outside of the European Union, so we have to conduct deer surveillance throughout the EU at a cost of so much’, nobody complies.” (pers. comm., interview 19). For them, going beyond current surveillance has no chance of happening because of the minority of Nordic countries represented on the board. The problem is that the wider the surveillance, the more cases might be found, increasing knowledge of the prevalence in Northern Europe but without any possible comparison with the situation in countries that did not undertake this survey. Moreover, surveillance without cases does not mean that the territory is CWD-free, but depends on how much effort is put into the process, and what parts of the populations are tested (slaughtered, road kills, dead found, young/old, etc.). There is now a program for establishing freedom of CWD in Norway [46].

The importance of reindeer and moose in local and Indigenous economies in Nordic countries can explain the positions of different European states. A crucial step could be determining the zoonotic potential of CWD [36,47,48,49,50,51], but because of contradictory results in North America and poor data in Europe no conclusion is possible yet. However, there is no evidence of zoonosis so far [1,2,4,10,36,47,52]. Many studies are going on and the results could have an impact on the development of research on CWD in the future because with “an obvious zoonotic potential, there would be much more money available for CWD than there is now” (pers. comm., interview 4). Also, the zoonotic potential would be related to the exposure of some populations. More precisely, Indigenous populations and local hunters from North America and Nordic countries are used to consuming cervid meat in their diet and could be more exposed to CWD than any other human populations.

### 3.7. How Are the Indigenous Sami Herders Participating in the Surveillance?

In the USA there is a genuine difficulty in reaching Indigenous people in terms of CWD management because of marginalized communities and a strained relationship with the federal authorities, but their role could yet be essential in the management of the disease (pers. comm., interviews 4, 5). In Canada, the involvement of Indigenous communities in the management of CWD is more developed [14,16]

During our study, we identified a close interdependence between two groups: the scientists and the locals. On the one hand, because clinical signs in the field are not sufficient to detect the prion, identifying CWD relies on laboratories to analyze the samples from cervids (pers. comm., interviews 1, 3). On the other hand, it is not possible to collect samples without the local hunters and herders. Therefore, diagnosis requires a combination of these two interlinked activities.

In Sweden, since 2018, a big step was to integrate the Sami on the board of risk management because reindeer herding is in their hands. The Swedish Sami Association (SSR) is the main Sami point of contact for the Swedish authorities since it is an organization that represents Sami herders and promotes Sami interests. Its role is to act as the link between the herders and the authorities, to communicate to the herders the measures and updates on the disease but also to educate the people who organize the surveillance about reindeer husbandry (pers. comm., interviews 3, 17, 18). SSR became a central partner for CWD management and herders’ integration into the survey in Sweden. This was confirmed during the interviews we undertook: all the herders we met were well informed about the disease and were on the frontline of the surveillance process (pers. comm., interviews 1–20).

However, at the local level, the people have their own logic. Analyzing the coordination of individuals for the management of avian influenza, Figuié [19] showed that people’s priorities are “to protect their immediate interests (e.g., avoid the slaughter of their flock)” and “to remove them from the grip of the authorities (national, international, public, private)”. Considering the participation of Sami herders in the surveillance, sampling is not an insignificant act because it can result in finding cases in the herd and potentially to culling, which can explain why not all herders are always enthusiastic, even more with a disease that they cannot see for themselves (pers. comm., interviews 1, 3). However, all the herders we talked to were in favor of sampling and saw it as a way to prove they have healthy animals: “Even if we, the herders, are convinced that there is no CWD in our herds, we need to convince the general public and the only way to do that is with more testing” (pers. comm., interview 12).

Herders seem to prefer to sample by themselves instead of being forced to do it by the authorities, but this is associated with several difficulties (pers. comm., interviews 3, 7, 9, 12, 16, 18). One of them is the difficulty in sampling at-risk animals over vast regions:

“It is a struggle to find them, if they die in the forest, depending on where in the country […] but it is also finding them and going back with a knife, and cutting the head off, and collecting a sample, it is quite a lot to demand. […] We also have practical barriers: during the winter it is very cold and dark, and we have the distances, it can actually take hours.”

(pers. comm., interview 3)

For them to take the sample is pretty fast, but the associated activities (packing the sample, filling in paperwork and mailing it) can be time-consuming, and the costs of it are never fully covered (pers. comm., interviews 12, 16, 18). Sampling can also be a problem on a cultural level. For the herders, finding a dead reindeer involves emotions which can contribute to making the sample collection difficult. One herder told us that when facing a dead reindeer, despite training and communication, it is always harder than it looks on paper (pers. comm., interview 18). Nevertheless, it seems that the collective and individual interests overcome these difficulties, and one herder explained that they have “to consider the bigger picture”, because by sampling a few animals per Sami community, they “protect thousands of animals, you have to always remind yourself of the bigger picture” (pers. comm., interview 12).

It seems, therefore, that the participation of the Sami herders in the surveillance process is in line with their own wishes, although we might have expected herders to demand greater involvement.

### 3.8. A Sami Alignment with the Governments’ Position: For How Long?

Our results show that since 2016, both Sami herders and their representative, SSR, have followed the Swedish state in the management of the CWD and that, for now, they see the sampling process as a way to prove that their herds are healthy. Hence, the current stance can be seen as an alignment with the measures put in place by authorities, a rather unusual situation in the colonial history of Sami–Swedish state relationships.

The controversy about the recognition of a classical and an atypical CWD is fundamental to understanding the Sami alignment and the management of the CWD risk in Sweden. All the herders we talked with said similar things: “In the beginning, we were really worried but because we didn’t detect any CWD, it was a relief. Now, we know there is no CWD” (pers. comm., interview 11). At the time of the interviews, the herders were not worried about CWD due to the high number of negative tests, and positive cases found only in older moose (pers. comm., interviews 7, 9, 11, 12, 13, 14, 18). Some of them think that the ‘atypical’ CWD had already been in Sápmi for a very long time, and has just been discovered lately, meaning that it is not a threat to reindeer husbandry (pers. comm., interviews 1, 13, 16), a hypothesis that is neither validated nor refuted (pers. comm., interview 1). For the representative of SSR, this would also explain why CWD is not spreading among reindeer herds, in spite of a moose case in the region:

“The reindeer herders work very closely with their reindeer, I can’t say for sure, but as a lot of people say, if there were to be any spread of CWD right now, in a massive outbreak, we would know it. Perhaps, we wouldn’t know it is CWD, but we would know it is happening.”

(pers. comm., interview 18)

Our interviews with reindeer herders also showed that CWD was not considered as the major current problem, especially since it has not been detected in any herd (pers. comm., interviews 7, 9, 11, 12, 13, 14, 18). The representative of SSR explained that there are many other threats considered worse for the reindeer herders (long springs, bad winters, etc.) (pers. comm., interview 18). Thus, the fact that CWD is not seen as a priority depends on the ‘level of threat’ perceived at the present moment. Another important point we observed among herders about CWD is fatalism. The fact that a herd contaminated by CWD cannot be cured, and will probably not escape slaughter gives them the idea that there is nothing to be done. As a result, it was challenging to the stakeholders we interviewed to imagine anything beyond surveillance.

**Figure 2:**
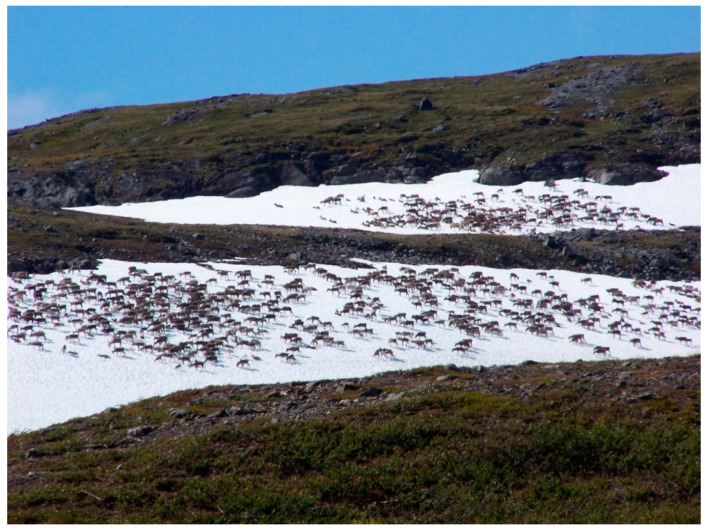
Free-ranging reindeer in their summer grazing lands, in Sarek National Park, Sweden. Source: S. Roturier, July 2009.

Even though the atypical CWD hypothesis reassures reindeer herders, they are obviously worried about the possible consequences of CWD cases in their herds. The culling in Nordfjella created a dramatic precedent for them, a sword of Damocles poised over their herds. Beyond reindeer herders, all the stakeholders we interviewed agreed on the fact that, if it happens, the impact of CWD on reindeer herding would be catastrophic for the reindeer herding industry and for Sami culture. CWD in Sami reindeer husbandry would have disastrous outcomes. A herder with an infected reindeer would lose all his herd, including all the reindeer of his family and other members of the community, and would probably have to quit this activity because of the possible contamination of the environment and the difficulty of starting a new herd (pers. comm., interview 6, 7, 12, 13). Also, for the herders we talked to, a case in reindeer herding might mean that it is already too late because the reindeer mix with each other and move a lot (Figure 2). For them, a case would imply existing spread of the disease (pers. comm., interviews 7, 9, 11, 12, 13, 14, 18). For the SSR representative, the Nordfjella strategy consisting of ‘cleaning’ an area of CWD would not work in the reindeer herding context because of the herds mixing with each other, especially in summer, and because of seasonal migrations (pers. comm., interview 18).

At the time of writing, no CWD case has been found in Sami reindeer herds and we believe the Sami alignment with the state is strongly related to this situation. Surveillance has the purpose of proving that herds are healthy. Positive cases in the future would probably modify this trust relationship, depending on the subsequent strategy (massive culling or not) and the social and cultural considerations in the measures.

### 3.9. Managing CWD in a Colonial Context

We have shown that managing the CWD risk in northern Fennoscandia, or Sápmi, involves the cooperation of different groups of stakeholders, among which the Sami have a key role. As shown by Figuié [19], the collective management of an emergent disease requires adherence to a common framing, which seems to have been the case for CWD between 2016 and 2020. During this time, all the stakeholders, including the Sami herders, have adhered to the sampling strategy, and to some extent to the culling in Nordfjella. However, she also showed that defining a common interest is a prerequisite to collective management (14). In other words: “it means to choose what is most important to preserve, to defend or where to go” (pers. comm., interview 5). Defining a common interest can lead to difficulties during a health crisis: depending on the stages of the crisis, priorities, constraints, values or ethics may vary between stakeholders [19,53]. Indigenous Sami herders, scientists, administrations, or states can all claim their own interests as being legitimate, but from different sources: Indigenousness, science, public health, and democracy for instance. Defining a common interest can thus be challenging because it relies on power relationships between stakeholders and the possible hierarchy between the various interests, especially in a colonial context. It is thus essential to analyze who is speaking the truth about the existence of a common interest [19,54].

We identified three unbalanced relationships that could possibly lead to a misalignment of reindeer herders from the current common interest. The first one is between Sami herders and scientists. Being herders from a young age and for generations, Sami herders possess a deep ecological knowledge of the reindeer and of the environment on which they depend for their livelihood. This body of knowledge, including practices and worldview, is critical to managing CWD, since it remains inaccessible to science and veterinarians and epidemiologists. Scientists are well aware that CWD management depends on Indigenous knowledge. One veterinarian argued:

“When we spoke to the Swedish Board of Agriculture, they wanted strict areas, like ‘You must tell us the size of the zone’, and we said ‘When it happens, we need to speak to the people to understand the cervid population in the area’.”

(pers. comm., interview 3)

According to this individual, the idea of gathering knowledge in order to understand the disease and before any action was central:

“We need experts knowing about reindeer, and the structural area. So, reindeer herders can be the experts because they know what is going on. You would ask them to get information. To know to whom the reindeer belongs, if they have been moving animals, if it is an area with other reindeer close.”

(pers. comm., interview 3)

However, the Indigenous knowledge-science dialogue is not straightforward, and most of the time occurs at the expense of the former [55]. During discussion processes with dominant institutions, Indigenous people have to adapt their language and worldview to those institutions and never the opposite [56,57]. It alters the way of thinking about the relationship to the animal for instance, the consequences of the disease (from a human or non-human perspective), and the language of action [53]. This is totally applicable to health risk management. Medical and veterinary sciences generally show “little interest in the rationalities of the actors involved, the heterogeneous nature of their interests and the collective determinants of individual behavior” [19]. In the case of CWD, a largely unknown disease, risks of disagreement between science and herders’ knowledge, and subsequent misalignment are high, especially in a situation where the Nordfjella culling happened. Even though all the scientists we have interviewed were in favor of Indigenous participation in the surveillance process, and were aware of the importance of Indigenous expertise, their results are produced and used by the authorities, translated into scientific language, and will serve their decisions with or without the consent of the Indigenous people.

The second unbalanced relationship is between the Sami and the Swedish state; this is grounded in a colonial history including marginalization and dispossession based on State decisions. As shown in another context [17], herders exchanged a lot of information about their cattle’s health within an informal network of trust, which the authorities cannot access. For the authorities it is virtually impossible to integrate such networks because of mistrust and the colonial history (pers. comm., interviews 7, 9, 11, 12, 13, 14, 16, 17, 18). For instance, to Sami herders, SBA clearly symbolizes central authorities. To let the national agencies define the common interest is thus viewed as tricky. In addition, identifying a national interest means hiding Indigenous particularities [58] and raises unresolved issues such as the place of Sami reindeer herding, and Sami culture in general, in Swedish society. Herders could agree with a common objective—to fight a disease—but disagree on the measures imposed by the authorities [19,22]. Or they could act in a different way from the authorities to reach a common goal. Gradually, a misalignment might appear if the Sami consider that the policies are no longer in their interest [19,22]. Nevertheless, the new strategies of the states will have to be supported through the trust and mutual aid network of herders [59].

The third unbalanced relationship occurs at the EU level and demonstrates what little power the Sami have with respect to such an issue. Managing health risk at the EU scale automatically implies the homogenization of realities in order to fit with European objectives and priorities, and not local ones. The hierarchies in defining common interest, and thus priorities, are strengthened since the only ‘competent’ authorities in Sweden are the main interlocutors to the EU Commission. Therefore, defining a common interest will depend on the national institutions’ interests and on their relationships with Sami herding. Our interlocutors at the commission stated that meeting the Sami herders on this matter would only be informative to understand local difficulties but probably not sufficient to change any direction (pers. comm., interview 19), even though the Sami are the only recognized Indigenous people of continental Europe. They described a powerful mechanism:

“Let’s be very clear: when there is a health crisis the rest disappears. […] So, the response to a health risk can be very, very disproportionate because once public concern gets in, there is a political rush and public pressure makes the measures extreme. And to say, at that point, ‘we want to protect the Sami, they are an Indigenous people of Europe’. No, in practice, it doesn’t happen.”

(pers. comm., interview 19)

In other words, it is difficult to address other priorities in a health risk context. The commission thinks in fragmented departments, and the health risk disease section has to think about the health of consumers first. Our analysis actually raises the idea of integrating cultural issues in food safety authority competencies, or at least to increase their place in the decision-making [60].

The surveillance program has the purpose of proving that reindeer herds for the Sami, cervid populations for the scientists and region for the Swedish state are ‘healthy’. However, as soon as it reveals a different situation, the position of the herders regarding these measures and their efficiency could change significantly, and generate a misalignment. This misalignment could result in mistrust in the authorities because of interests that are not shared, a Sami opposition to massive culling, the end of cooperation in sampling (and thus the interdependence between herders and scientists), or the collapse of the herders’ informal network (because of the fear of positive cases in neighboring herds) in favor of a more individualistic way of working.

In this study, we analyzed the time sequence from March 2016 to August 2020. Retrospectively, our fieldwork occurred during a relatively calm period, from an epidemic perspective, and depending on the next event, CWD management could move to another phase. We thought that five scenarios were possible after this period: a new case in a moose, a new case in a wild reindeer, a new case in another species, a first case in reindeer herding, or no new cases. In our interviews, we understood that the Nordfjella strategy of culling every single reindeer of the region in order to cut down the CWD spread was the cause of great hope for all the stakeholders. After the slaughter, the plan was to have this area “lie fallow” to try to get a CWD free environment before reintroducing reindeer five years later (pers. comm., interview 1). The results are still awaited in order to know if this worked (which would be a major step in the disease management at the international level), or if the prion survived in the region and will start a new epizootic (pers. comm., interviews 1, 3, 4, 5). However, on 10 September 2020, the Norwegian Veterinary Institute declared a new case of CWD in a wild reindeer from Hardangervidda. This was the first reindeer testing positive since 2018 (during the Nordfjella culling) and it is very likely, given the diagnosis from the Norwegian Veterinary Institute (saying that the material from a lymph node tested positive, but the brain tissue was negative), that there are other infected cases in the area. We do consider that this event strengthens the questions we raise in this paper, but it is too soon to have an idea about the changes it will lead to in the future. The only point we can highlight at the time of writing is that the hope for Nordfjella no longer seems valid, and containing the epizootic seems unlikely. It is a critical step in the CWD situation in Fennoscandia and it may accelerate the misalignment of the Sami herders because it means, at least, three things: the Nordfjella hope for containment of CWD can obviously no longer exist; CWD keeps spreading in the South of Norway; and the authorities failed to stop it.

It appears more crucial than ever to increase collaboration with the Sami reindeer herding community to ensure that their interests will be taken into account in the decision-making processes and the measures that will be implemented afterwards. The new case in Hardangervidda brings even more uncertainty about the spread of CWD. Further research must include all possible knowledge available, including Indigenous Sami knowledge (e.g., [44]), to provide democratic management, and avoid the future of Sami reindeer herding being controlled by CWD management.

### 3.10. “Reindeer Herding with CWD Is no Reindeer Herding at all”: From the Prion to the Pastoral System

Today Sami reindeer herding has to face many threats, including a wide range of land encroachments and these coincide with climate change. CWD could weaken reindeer husbandry, the associated livelihood and culture even more deeply (pers. comm., interview 18) [61,62]. However, for one Sami academic,

“Reindeer herding will not stop because of CWD but the number of reindeer and the number of herders will be limited by food, climate change, extractive industries, but we will have reindeer herding”.

(pers. comm., interview 6)

Thus, the question is: in what form?

**Figure 3:**
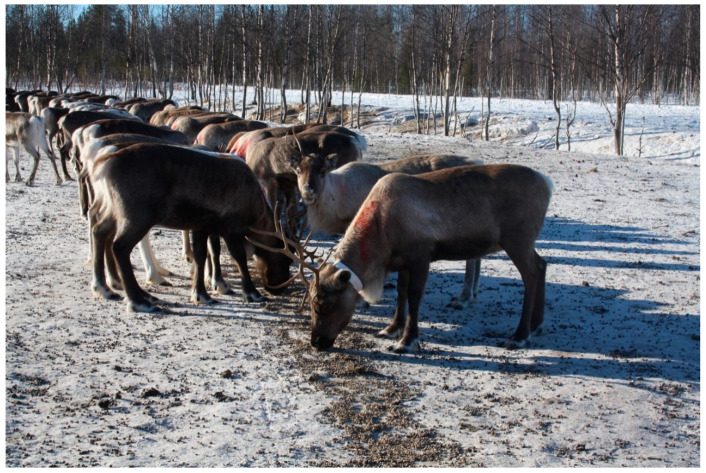
Reindeer consuming feed brought into their winter grazing lands in Pajala, Sweden. Herders can supply reindeer with industrial pellets or silage to avoid them starving. Reindeer can be kept in corrals or be fed in the forest too. Source: A. Poiret, March 2019.

According to the interviews, CWD could be a factor in increased pressure to turn from a pastoral towards to farming-driven model (pers. comm., interviews 6, 7, 9, 11, 12, 13, 14, 16, 18). Reindeer pastoralism has changed a great deal since the 16th century and will keep evolving. It has been mechanized, partly sedentarized, and has adopted and adapted to new technology. An important change during the last decade has been the use of artificial feeding to provide emergency rations in winter (Figure 3) because of land encroachment and climate change-induced poor grazing conditions [63]. These are important costs for the herders, and could also modify the behavior of reindeer (pers. comm., interview 6). All herders we interviewed are reluctant to embrace this possible evolution because, according to them “[reindeer] are not made for that” (pers. comm., interview 12), and that would represent new dangers. From a health perspective, to keep reindeer in corrals to feed them will automatically increase diseases because of a higher animal density (pers. comm., interviews 6, 16, 18). With CWD in grazing lands, or massive culling, the herder would have to feed their reindeer all year long. The bad winters have already changed the behavior of reindeer and the conditions of herding, and CWD management could accelerate the move. Going towards a farming model would also have dramatic consequences with the loss of the pastoralist culture, practices and knowledge that will be irreversible (pers. comm., interview 6). Sweden and the EU are not actively pushing for reindeer farms, but the possibility is raised by CWD (pers. comm., interviews 17, 19). This is not surprising since health crises are opportunities for authorities to interfere and make changes of various kinds [19,21,64], including in pastoral, nomad societies that often escape state control [58,65].

Reindeer herding, as one of the last nomadic pastoral systems carried out by an Indigenous people, is unique compared to other livestock production in Europe. On this basis, it escapes some EU rules. One example is that there is no animal registration [66]. In a context of health risk management, this can be seen as an unjustified irregularity hindering traceability of animals due to the lack of data. The number of reindeer in herding communities has also been a matter of dispute with all Nordic states for centuries and they may wish to interfere in Sami affairs by introducing more control in Sápmi in order to regulate the activity (pers. comm., interviews 7, 9, 16, 18) for epidemiologic reasons. According to one veterinarian we interviewed:

“It is really complex, this is why for other species, you have baseline data to try to track and understand, that is for the disease tracing, that is to try to figure it out all of that, and with the reindeers we don’t have this system. So, it is a challenge, and you have not only the gathering but also the transport.”

(pers. comm., interview 3)

In many contexts of diseases or virus transmission, transhumance is also posited as a vector of epizootics [59,67,68]. With CWD, transhumance can be seen as a concern because of the ability of the prion to survive and to infect the environment in the locations to which the herds move. With the Nordfjella cases, the EU took measures at borders to stop animal movements with Norway, but it exempted reindeer, which were able to cross the borders between Norway, Sweden, and Finland (pers. comm., interview 19). To our interlocutors at the EU Commission, this decision was a mistake because “it was done in order to respect traditions, but in practice, healthy animals were exposed to grazing areas where there may have been contaminated animals. […] So, it is certainly not a thoughtful choice in the medium to long term” (pers. comm., interview 19).

Disease risk management entails trade-offs between health issues and socio-economic and politico-cultural issues, and a health crisis exacerbates the former at the expense of the others. The poor understanding of Sami reindeer herding in state administrations could be particularly detrimental for reindeer pastoralism. For example, during this interview, our interlocutors from the Commission said

“The Sami have to accept that movement may no longer be the best thing to do and that animals, when they are healthy, stay in an area that is known to be uncontaminated, and that this protects their own herds. […] As long as we have semi-wild or wild animals, it is extremely difficult to control this type of disease.”

(pers. comm., interview 19)

To them in the context of CWD, transhumance—i.e., the very essence of reindeer herding—increases the risks to animals and the livelihood (Figure 2). By opposing health issues with Sami culture, there is a risk of targeting the debate on the wrong problem. Looking for the complementary elements of the two values might actually be more efficient. Transhumance might thus become a great issue with respect to avoiding diseases in general, unlike fenced cattle [67]. It is thus a political and ideological choice as much as an epidemiological one.

In Sweden, extensive reindeer husbandry is protected by the Reindeer Husbandry Act (*Renskötselrätt*) but is also contested by other land users. The fact that ancestral rights are related to reindeer herding makes this activity a key pillar of the power relationship in the land use in Sápmi. Prohibiting transhumance or free grazing for health reasons would be a major shift in the geopolitics of the territory. According to the Sami academic we interviewed, it would be a colonial process to make the reindeer domestic, fed like cattle in corrals, modifying their behavior, affecting herders’ livelihoods, and making room for other land uses (pers. comm., interview 6). It would definitely change the power relationship between herders and other land users: “If you don’t have reindeer husbandry in the area, you don’t have to give them space to live” said a herder, afraid to lose pasture lands to extractive companies (pers. comm., interview 13). Because reindeer grazing lands cover the entire region, from bare mountains to coastal conifer forests, the Reindeer Husbandry Act protects the land from extractive projects [69,70,71]. Removing reindeer from the landscape through a grazing ban, or worse culling, to decontaminate the environment or the herds would also imply legal issues (pers. comm., interviews 6, 12, 18). According to our contacts at the Sami parliament of Sweden, their rights (*urminnes hävd*) are based on activities since time immemorial, so a temporary gap in a continuous use of a land should not erase them. However, it is very difficult to predict how it would affect the balance of power in politics, land use negotiations or media treatment (pers. comm., interview 20). Even if the rights are maintained, having no reindeer in a grazing area could weaken the position of the herders within the territory, and accustom land planners and owners to a reindeer-free landscape.

CWD could start a cascading effect that complexifies the already tense situation of threatened reindeer herding. It could lead to removing reindeer from entire grazing areas, temporarily or permanently. Besides cultural, legal, and political issues, this would have consequences for the development of other land encroachment activities. It would also generate many transformations regarding subarctic ecosystems by removing herbivory, but also prey for large predators. Locally, it could lower husbandry options, adversely affect Sami culture, and degrade the socio-economic conditions for the Sami society for the sake of a health problem.

### 3.11. The End of the Transaction between Indigenous Herders and Reindeer?

In a famous article, the anthropologist Tim Ingold analyzed the relationship between human and reindeer through the complex and evolving process of domestication [72]. The various forms of reindeer pastoralism experienced in Sápmi over the centuries were the result of the evolution of technology but within what he called a transactional relationship, where both human and reindeer have their own interest in reindeer husbandry (i.e., a form of social contract between the two parties). We believe that this relationship has contributed to protecting reindeer pastoralism from health crises so far, and we argue that CWD has already modified the human-reindeer transaction.

The work of the anthropologist Frédéric Keck on avian flu in Asia showed that, regardless of the degree of virulence of a disease, and whether the pandemic actually happened or not, the new measures, health standards, fears, and imagined scenario create a new reality. CWD in Sápmi means that Sami husbandry and the transaction between herders and reindeer will change with or without CWD cases in reindeer herding because CWD is now part of the concept of reindeer husbandry. In other words, with or without a CWD outbreak in reindeer herds, the CWD political crisis has already happened and will have long-lasting consequences for Sami herders. Canguilhem explains that any pandemic results in “the loss of biological innocence” by transforming our reality with new standards and creating “an irreversibility of biological normativity” [64]. This new normativity thus changes the nature of the transaction. Producing reindeer meat in farms is a way to assure consumers that there is no risk, and at the same time to avoid contamination from other herds. However, as a result, the contract between human and reindeer described by Ingold, where the herder provides security to the reindeer and the reindeer guarantees subsistence to the herder, becomes a relationship of surveillance, mistrust in the reindeer (potentially sick), and fear of the herder (source of intense stress).

The move towards artificial feeding and fenced herds has already started in order to adapt to climate change, save herds from starvation, and maintain the herding economy [25,73,74]. A further development towards reindeer farms would mean losing the link between the animal and the herder in favor of a more controlled and technicized structure and to follow the trend of industrial production of animals, which started in the 19th century in other livestock production [75]. However, for the herders, moving to intensive reindeer herding does not make sense since the reindeer are currently healthy: there are very few health issues in reindeer herding today and across history (pers. comm., interview 13). When we interviewed herders, they did not recall any significant epizooty in the history of Sami reindeer husbandry (which does not mean that there was no epizooty earlier in the region). The herders do not really feel confronted by diseases in their daily work:

“[w]e keep reindeer on such large areas, so it is like self-selection, the reindeer who are sick and so on, they will die, they will go away. So, they get naturally sold out in some way.”

(pers. comm., interview 13)

Now, with the use of winter corrals, artificial feeding and motorized transportation (due to traditional migration routes being disturbed, especially by dammed rivers), new diseases are appearing, and herders have to adapt. Traditionally, when a crisis occurred, the nomadic families would disperse over the territory to keep a distance between each clan and their reindeer (pers. comm., interview 6). However, nowadays, such an option is impossible due to the decrease in grazing lands. Surprisingly, the opposite strategy seems to be the recommended health standard (pers. comm., interview 19), despite the fact that sedentarization and high animal density favor disease transmission [65]. Hence reindeer farms, with closer animal contacts in smaller spaces would worsen health conditions for the herds with new diseases and new remedies (vaccines, antibiotics, etc.).

We believe that the process affecting reindeer herding is in fact in opposition to contemporary changes in animal ethics in Western societies. Indeed, this separation in the relationship between herder and animals, in response to a health crisis, has ontological implications because it questions more broadly the relationship between humans and animals [53]. On the one hand, reindeer husbandry is forced to gradually move towards an industrial model, and on the other hand, consumers are increasingly advocating a model that resembles current reindeer husbandry. There is therefore a time lag between the application of measures dictated by the authorities and the values advocated by public opinion. It is an issue of priorities between animal welfare and ethics or a utilitarian conception of reindeer herding. It is also a matter of borders between human and non-human, wild and domestic, that can be softer—with zoonotic potential—or strengthened with culling policies [76].

Nordfjella culling transposed to reindeer herding is clearly the elephant in the room in CWD management today, and none of the interviewees could react to it. Obviously, massive culling is a divisive matter. First of all, it has not been proven that it actually worked since the disease keeps expanding despite the slaughter. Keck explains that various strategies exist to address the threat of a pandemic: a “make-live” approach (vaccination, simulations), a “let-die” approach (with neglected diseases or populations that don’t mobilize) and a “make-die” approach (quarantine, slaughter) but rarely, the “let-live” approach is considered [77]. Regulation does not require the culling of herds and, even in Norway, the authorities only slaughtered the cervids with “classical” CWD in Nordfjella (until September 2020, the “classical” CWD was only found in Nordfjella, it will be interesting to see what decisions will be taken following the new identified case in Hardangervidda). Massive general slaughter is seen as impossible for political reasons. The path of the let-live approach should be explored. It could, for instance, consist of “removing [the animals] from the market economy that causes their diseases” [18] and inspired by decisions taken in relation to reindeer herding after the Chernobyl disaster. Keeping the transaction as it is might, therefore, be a means to resist diseases and potentially intrusive and authoritarian management in a colonial context like Sápmi.

### 3.12. Disease Risk Management: A Key Process in the Distribution of Power

We have seen that CWD is far from a simple biological and epidemiological issue but a political and cultural one that has generated various processes at various scales. With all these elements, we consider that CWD risk management can lead to several possible futures regarding a reconfiguration of governance in Sápmi.

The health risk management could be based on knowledge and skill-sharing according to the complementarity of the stakeholders and on real distribution of power. By integrating other knowledge, visions, values, and concerns in the decision-making process, the risk management can take a more “democratic turn” [19,78]. This is the entrance of “profanes” into “hybrid forums” (and not only based on Western scientific experts) or co-management [79]. This new organization could lead to new systems of values and structures, a domain in which Indigenous and local knowledge can be particularly useful [80]. For instance, new surveillance measures could become critical tools against the commodification of the living and the animal conditions in various husbandries [18].

We believe that an essential element is that the nature of the relationship between the Sami herders and the Swedish state on the CWD issue during 2016–2020 is based on an efficient interaction. There is a common interest in not having CWD for both groups. This dimension is quite rare in the Sami–state relationship because of multiple tense situations in Sápmi [69,81,82]. The SSR representative explained they have a good relationship with the state regarding CWD. According to her, the herders have been involved from the beginning of the process, which is unusual, and are considered by the people from the state as important to work with in order to find a solution (pers. comm., interview 18). She added “they understand that they don’t understand” the reindeer herding (pers. comm., interview 18). The herders we interviewed were satisfied with the surveillance process and their involvement, and considered that their knowledge has been respected so far (pers. comm., interviews 7, 9, 11, 12, 13, 14, 18). On the authorities’ side, the same sentiment was expressed: the relationship with respect to CWD is good and new, since reindeer are generally in very good health, the ‘animal diseases’ section of the Swedish Board of Agriculture (SBA) usually has little interaction with the Sami. However, the cooperation happens but remains fragile. The authorities explained that it is difficult to convince the herders that there is no hidden agenda behind the CWD management.

However, on the herders’ side, there is a clear mistrust regarding this relationship in the future. There is the fear that the Sami communities are giving away information that could be used against them. For instance, giving information on reindeer movements for CWD management could be used by the government in other contexts (pers. comm., interview 18). It is also fundamental to position this relationship in a political and balance of power context: the national authorities have much greater decision-making power than the herders. Therefore, the cooperation between the Sami and the state is structured as an asymmetric dialogue.

The health risk management can also cause a strengthening of hierarchies that leads to drastic and vertical decisions. First, the dominant disease studies are “confined research”, undertaken in laboratories [79]. It is thus difficult to be part of a genuine active dialogue with local stakeholders and to take an interest in alternative concepts and methods [79]. This confined research may symbolize a form of effective discontinuity between the field and the scientific results, and this can accentuate the local feeling of being dispossessed of the process. Again, in Sápmi, the herders did not express this idea and felt sufficiently involved. However, an involvement of local stakeholders does not automatically mean a better distribution of power, especially during an epidemic outbreak, although maybe it signifies the end of the monopoly of scientists and technocrats’ knowledge on health risk management in Fennoscandia. It could lead to new practices and norms being internalized by local stakeholders, perpetuating existing hierarchies, but with new forms [19,83,84]. The integration of the Sami in the surveillance process did not mean that the position of Sami knowledge changed in the hierarchy of knowledge, it did mean that they were taken into account in a form that must be adapted to Western scientific standards, language and thinking and above all with no guarantee of involvement in the decision-making process.

Whatever the direction it takes, CWD might lead to political transformations in Sápmi and in Fennoscandia. There are inclusive and cooperative processes aimed at the Indigenous Sami herders, but this study showed that they take place in a westernized structure of health risk management. The evolution of the surveillance program and the implementation of the various measures to fight CWD, so far more than CWD itself, will have tremendous consequences for the evolution of Sami reindeer herding and have to be understood in that context in the future.

## 4. Conclusions

In September 2020, the identification of a new CWD case in a wild reindeer in Norway started a new episode in the disease management in Fennoscandia. This paper presents various questions that are relevant when considering this new step in this not only epidemiological but socio-cultural and political crisis. It is important to situate CWD in its political and historical context, explaining the interests in the participation of the Sami in the surveillance program. One element that emerged from this study is the controversial distinction between classical and atypical CWD, and its influence on the behavior of the herders who were, at the time of the interviews, not worried about CWD. However, the disease is a sword of Damocles hanging over reindeer herding, since cases in Sami herds would be a catastrophe. This all takes place in an already tense context with reindeer husbandry experiencing many threats, and gradually heading towards more intensive practices. CWD would have major impacts on the evolution of this activity and its modalities. Health standards and cultural practices can have contradictory interests, and the situation creates a need to define priorities on which the involved stakeholders should act. However, these new guidelines will depend on the power structures in place and the related dominance. Because of the role of reindeer herding in Sápmi, the prion disease would also generate a shift in the organization of the territory. We have noticed that, even without cases in the reindeer herding area, CWD has already influenced the way of seeing the Indigenous activity when establishing the new health standards. This cascading effect could also lead to a change in the relationship between the herder and the reindeer. CWD could result in various futures with respect to land use, traditional practices, ancestral rights, cultural continuity, and balance of power. We believe that the issues identified in this paper are transposable, with their own specificities, to other territories and contexts. Similar work could be developed with the Indigenous communities in the USA or Canada to study the impact of CWD and sanitary measures on their ancestral practices (relationship to the animal, relationship to the land, exposure to the prion) and the level of participation in such processes.

The evolution of diseases is uncertain. This makes a diversity of expertise necessary in the construction of knowledge. For that reason, we consider that it is crucial to have a social approach to accompany biological and veterinary studies of CWD in order to politicize the health question and to analyze it in relation to dominance, in particular with marginalized populations. The new reindeer case in 2020 in Norway may bring an acceleration of the measures implemented and the situation of emergency even closer. An emergency situation usually leads to emergency decisions, which finally tend to become anchored in the sanitary landscape and which will irreversibly modify Sami reindeer husbandry.

## Figures and Tables

**Table 1:** List of the interviews, dates, and location of the interviewees. All the interviewees were anonymized.

N°	Date	Place	Interviewee’s Institution	Function
1	26 May 2020	Oslo, NOR	Norwegian Veterinary Institute	Veterinarian
2	26 May 2020	Paris, FRA	Retired	Veterinarian
3	2 May 2020	Stockholm, SWE	Swedish Veterinary Institute	Veterinarian
4	3 June 2020	Ithaca, NY, USA	Cornell University–College of Veterinary Medicine	Assistant research professor
5	12 June 2020	Madison, WY, USA	US National Wildlife Health Center	Emerging disease coordinator
6	26 June 2020	Tromsø, NOR	The Arctic University of Norway	Academic Director of Sámi Research center
7	26 June 2020	Jokkmokk, SWE	Sirges Reindeer Herding Community (RHC)	Reindeer herder
8	29 June 2020	Jokkmokk, SWE	County of Norrbotten	Jurist
9	30 June 2020	Jokkmokk, SWE	Sirges RHC	Reindeer herder
10	30 June 2020	Stockholm, SWE	Swedish Environmental Protection Agency	Wildlife manager
11	30 June 2020	Vilhelmina, SWE	Vilhelmina Norra RHC	Reindeer herder
12	30 June 2020	Arjeplog, SWE	Semisjaur Njarg RHC	Reindeer herder
13	1 July 2020	Ammarnäs, SWE	Gran RHC	Reindeer herder
14	2 July 2020	Norrland County, NOR	Skjomen reindeer herding district	Reindeer herder
15	3 July 2020	Helsinki, FIN	Finish Ministry of Agriculture	Jurist
16	7 July 2020	Uppsala, SWE	/	Veterinarian
17	8 July 2020	Stockholm, SWE	Swedish Agency of agriculture	Veterinary inspector
18	8 July 2020	Umeå, SWE	Swedish National Sami Association	Reindeer herder
19	24 July 2020	Bruxelles, BEL	European Commission	Veterinary inspectors
20	20 August 2020	Kiruna, SWE	Swedish Sami Parliament	Jurist

## Data Availability

Not applicable.
